# Rheumatismus, or Rheumatism

**Published:** 1838-04-25

**Authors:** N. Chapman

**Affiliations:** Professor of the Theory and Practice of Physic, in the University of Pennsylvania


### THE MEDICAL EXAMINER.

DEVOTED TO MEDICINE, SURGERY, AND THE COLLATERAL SCIENCES.

EDITED BY J. B. BIDDLE, M. D. AND M. CLYMER, M. D.

No. 9.] PHILADELPHIA, WEDNESDAY, APRIL 25, 1838. [Vol. I.

### RHEUMATISMUS, OR RHEUMATISM.

*By* N. Chapman, M. D., *Professor of the Theory and Practice of Physic, in the University of Pennsylvania.*

[Reported for the Medical Examiner.]

It was remarked, on a preceding occasion, that the term arthritis embraced, originally, every inflammation of the joints, however differing in their causes or nature. But in the process of time, it came to be restricted to gout, exclusively, and that of rheumatismus, or rheumatism, was invented to designate another affection, having an analogy to, though not believed to be identical with it, which is now presented for consideration. Exactly the same objection applies to this term as to that of gout, each proceeding from a crude and erroneous pathological view. The derivation of the former has been given, and the latter comes from *rheuma*, a peculiar sort of humour, which, as was thought, being distilled into the joints, irritated them into inflammation. Exposed to the "peltings of the pitiless storm," old Lear, among other infirmities, is made to complain of "joint racking rheums." The credit of separating the two diseases, as specifically different, has been generally accorded to Sydenham, though without justice. Expressive of a morbid state of action, contradistinguished from that of arthritis, the term rheumatismus first occurs in Ballonius's treatise, "De Rheumatismo, et Pleuridite Dorsali," printed in 1642.

Like gout, this disease exhibits a great variety of aspects. The common nosological division of it is into acute and chronic, these being its leading and conspicuous states: and from its appearing, either in the joints or muscles, it has lately been subdivided into articular and muscular. Not embracing, however, the whole of the fibrous tissue, the term articular is defective. The disease, too, attacks the interior as well as the exterior of the body, no one of the contents of the great cavities, indeed, having a congenial texture, escaping. An adequate exposition of the disease, therefore, requires it to be presented in all these views, and each designated by some appropriate title. By far a better arrangement than any hitherto suggested, would be into fibrous and muscular rheumatism, each of which species to be considered in its acute, sub-acute, and chronic states, it being easy to bring under these heads every modification of the disease.

Of the varieties of rheumatism, the articulo-fibrous is the most common, and its principal seats are the large joints, one or more simultaneously affected, the shoulder, elbow, wrist, the knee and ankle, the fingers and toes being rarely implicated, though I have met with such instances.

By an accurate observer, we are told that, of an hundred and seventy cases of the disease, which he had noticed, only nine were exclusively muscular, and in thirty-three more, the muscles were engaged with the joints.*

An attack of acute inflammatory rheumatism may come on very suddenly, or more slowly, with a general soreness or rigidity, or directly by a chill, followed by the usual symptoms of fever, a very full, strong, round, or bounding pulse, hot skin, dry fauces, slighly furred tongue, and considerable thirst. The local affection, in some instances, anticipates the fever, and, perhaps, most commonly, such is the course of the disease. It may then be remarked, that the pain is steadily fixed at one point; whereas, when it is preceded by fever, it is fluctuating, attacking at once several joints, or changing very rapidly, from place to place. The stomach is little troubled, the bowels are constipated, the urine scanty, and very high colored, and without any sediment,—which, however, in the progress of the case, deposites lateritious matter, sometimes proving critical and salutary. The tongue ultimately becomes loaded with a thick, brown, tenacious mucus. Generally, with a tense skin, spontaneous clammy perspiration occurs, never from the part affected, however, having a peculiar acid odor, which, copious as it may be, does not mitigate the paroxysm, or in any way conduce to recovery. The fever is remittent. There is, for the most part, considerable alleviation during the day, with an exacerbation in the evening, attended, in some instances, by a vast increase of suffering, and especially if the patient be covered warmly in bed. This, which is the ordinary character of the disease, is sometimes considerably modified. Contracted in a miasmatic region, the influence of such a position is very discernible. The stomach here is greatly disordered, and copious vomitings of bile are common, the skin and eyes sallow, the tongue early coated with yellowish or brownish fur, the head aches, or is otherwise affected, and the fever is usually of the intermittent type.

In either case, the articular affection is pretty nearly the same, though perhaps less in the latter variety of the disease. The joint is red, tender, and very painful, particularly on motion, and ultimately becomes swollen and edematous, by which very sensible relief is afforded.

Every other portion of the fibrous membrane, however, may be the seat of an attack, even to the sclerotica of the eye, though it chiefly and most prominently occurs in the periosteum, locally, or more generally diffused. The sensation is that of a dull ache, or an acute, pungent, or darting pain, and, from its depth, is referred to the bone. It very often, too, is to be met with in the pericranium, especially on one side of the head, constituting hemicrania, and occasionally in the neurilema or fibrous integuments of the large nerves; the sciatic

* Haygarth's Clinical History of Rheumatism.

mostly, where it is recognised by singularly sharp pain, pursuing the course of the nerve.

Much of the preceding account is equally applicable to myositis or muscular rheumatism. It is, however, more apt to come on suddenly, or without any very significant premonition. Lumbago, especially, I have known to strike, as it were, like an electric shock. Three very remarkable cases of the kind were witnessed by me—the first, in drawing on a boot; the second, from an attempt to mount a horse; and the third, from stepping into a carriage,—each individual supposing, at the moment, that his back had been broken by a blow from behind.

Muscular rheumatism is mostly distinguished by a burning sensation, often severely painful, greatly exasperated by the action of the muscle, with swelling and redness of the integuments. The muscles, in any part of the body, may be the seat of it, though those of the back of the neck, of the sides of the thorax, or intercostals of the lumbar region and hip, are mostly liable to it, to which are applied respectively the terms, torticollis, pleurodynia, lumbago, and sciatica.

This form of the disease is singularly prone to metastases, either to other muscles, or the joints. But, instead of being confined to one or more muscles, it, on some occasions, displays itself over a wide surface, seemingly not much deeper than the skin itself, causing a painful degree of heat and soreness.

There is a further modification of muscular rheumatism, which claims here to be noticed, though hitherto differently assorted. My allusion is to those cases which so closely resemble tetanus, as to be deemed one of the varieties of that disease. The analogy, indeed, in some instances, is nearly complete. Commencing very much in the same way, we have ultimately violent spasms of the muscles, together with most of the other symptoms of that affection. But though, sometimes, this similitude is not uniformly presented, more commonly, perhaps, there is a partial or general rigidity of the external muscles, attended by a cold, collapsed, insensible surface, exceedingly feeble pulse, haggard countenance, and obtuseness of the senses and intellectual faculties. The condition, in a word, is that of torpor, as well of the body as of the mind. But, on reaction taking place, the sensations are very exquisite, and, with universal soreness of the muscles, tremendous spasms occasionally ensue.

Though these several forms of rheumatism, with the exception of the tetanoid, are generally febrile, there are occasionally acute attacks of the disease, attended by much pain, where vascular excitement is scarcely discernible. These are merely local affections in which, the general sympathies being quiescent, of course no constitutional disturbance arises. Exactly the reverse, however, sometimes happens; or the highest and most ardent fever prevails, independently of any outward or ostensible local affection; or, at all events, it is so trivial as not to account for such violent and extensive effects; and this is what is called rheumatic fever.

It has been usual to consider rheumatism as exclusively a centrifugal disease. True, in the main, it does not universally hold. The viscera, and every other structure, indeed, into which muscular or fibrous tissue enters, are, occasionally, liable to such attacks. I have witnessed them in the dura mater, in the diaphragm, in the stomach, or bowels, in the kidneys, in the uterus, and we have abundant proofs of its assailing the very fountain of the circulation. The heart is singularly liable to its aggressions, of which I have seen many instances, and still more are recorded. Bouillaud has, indeed, recently endeavoured to show a pretty constant coincidence between the articular and the cardiac affection, in its several diversities, of the recognition of which he arrogates the merit to himself. Except, however, as to the extent which, I suspect, is exaggerated, he has no just claim. The fact had been long determined, and taught by me, ever since my occupation of this chair, especially in tracing the etiology of the diseases of the heart, where it is assigned as a leading or principal cause.

Located in any of the internal parts, which it may be primarily, though much more commonly, by translation from the exterior, rheumatism bears so close an affinity to misplaced, or retrocedent gout, that it will be enough to refer to what was said on this subject. The metastasis is as sudden as in its kindred affections. On one occasion, I saw the translation in a moment, and without any premonition whatever.

No age, or sex, or condition, is entirely exempt from rheumatism. But it is mostly to be met with, in the acute form, about the meridian of life, rather in males than in females, and especially among the laboring classes, from, perhaps, their greater exposure to it causes; and in the lean or muscular, with a sanguineous temperament, than in the corpulent, nervous, or phlegmatic. Even very young children are liable to the disease. Examples of it I have seen so early as within the first year of life, usually in the muscles of the nape of the neck. It seldom occurs in aged persons, these being more subject to the chronic states of it.

Rheumatism depends on the sensible qualities of the atmosphere, and hence it is chiefly prevalent in positions greatly exposed to chilly moisture, and every where in the spring and fall, owing to greater and more sudden fluctuations of weather. But, at all times, by an exposure to cold, and particularly with moisture, or sleeping in a damp room, or damp sheets, or wearing damp clothes, or being thinly clad, or sitting in a draft of air, or entering ice houses or damp cellars, or other such places, an attack may be induced, and some of the worst cases I have ever encountered, were thus brought on in the midst of summer. Cold, operating on a previously heated system, is, indeed, so influential in the production of the disease, that the tetanoid variety of it, at least, is almost endemial in the torrid or intertropical climates. The writers of the West and East Indies speak emphatically of its prevalence, caused chiefly by sleeping, exposed to the dews and cool air of the night of those regions. Girdlestone, especially, mentions the remarkable fact of several hundred soldiers of a regiment being seized in this way, by sleeping in the open air, after a long and fatiguing day's march in some portion of India.

By Swediaur, Brodie, and other writers, rheumatism has been traced to a suppression of the gonorrheal discharge; or, at least, a connection is attempted to be made between the two affections, the one arising as the other declines. That inflammation of a joint has taken place, under such circumstances, I am not prepared to deny. But its origination in gonorrhea, or its identity with rheumatism, remains to be proved; and, at all events, on so imperfect a foundation, I am not disposed to erect a new species of the disease.

Not a little has been said of the predisposition to rheumatism, by some writers. That the circumstances mentioned, as sex, age, occupations, climate, and season, or states of weather, and temperament, do influence its production, cannot be doubted, though most of them rather as exciting, than predisposing causes. More than, perhaps, by any thing else, is a susceptibility to it created from the abuse of mercury. Yet a strong tendency to it is sometimes met with, pervading whole families, the occasion of which may be concealed, or certainly not always assignable to appreciable constitutional peculiarities. Examples are, indeed, given of its having been transmitted through successive generations as an inheritance, with the same obscurity in regard to the mode of its origin, or the condition by which its propagation is promoted.

With an unusual proclivity to rheumatism, an attack of it is readily excited by very slight causes, as a strain, or other injury to a joint, or by an indiscretion in diet, indulging freely in high seasoned food, or stimulating drinks; and in all instances, the liability to relapses is lively and enduring.

Much as gout sometimes resembles rheumatism, the prominent distinctions already given, with a careful comparison of the whole history of the two diseases, can scarcely ever fail to lead to a just diagnosis.

Excepting the two leading divisions of the disease itself, the fibrous and muscular, which are too clearly designated to be well confounded, it requires some attention to be enabled to discriminate, with precision, the modifications of rheumatism, as occasioned by its several locations.

As to this point, I have merely to state, that, a joint being affected, we shall find some marked difference in the symptoms; as the tendons, ligaments, and fasciæ, or the synovial capsule and bursæ, may be concerned. The pain, in the former, is acute and lancinating, the swelling slowly arises, and never attains to any height—is florid and exquisitely tender, comparatively seldom involving the surrounding textures. It is very much the reverse with the latter; or the pain is dull and steady, the intumescence quickly takes place, and is circumscribed and elastic, or pulpy, or edematous, from effusion into the adjacent cellular membrane; to which may be added, that the general constitutional disturbance is far greater in the first than second instance, in proportion to the degree of the local affection. In relation to the other cases, there is less difficulty, and they are, perhaps, sufficiently cognizable from what was said in the antecedent sketch of them, and I shall therefore decline a further prosecution of the inquiry into their peculiarities.

Certain spinal and neuralgic affections, have also a resemblance to some of the rheumatic modifications, and especially to lumbago and sciatica. Not now to go into any details, I have to observe, that, whenever the muscular system is concerned in these instances, and the pain is referable to the muscles restricted to them, and relieved by rest, and agonizing on the least motion. In the other lesions, without these characteristics, the source of the irritation is to be traced to a point in the vertebral column, betrayed by tenderness on pressure, from which emanate acute darting sensations, pursuing the course of a nerve or nerves. Not a little may also be learned by an inquiry into the causes, and mode of production, of these different affections.

Managed properly, acute rheumatism, excepting the tetanoid variety, can nearly always be cured, and never proves immediately dangerous, unless fixed in some important or vital organ. Neglected, however, or feebly treated, and especially where the local inflammation is violent and continues long, it so deranges the structure of the parts, as to irritate the system into hectic fever, followed by its wasting and debilitating consequences. Death in this way very often happens. But, though such may be one of its ordinary terminations, it is deserving of remark, that cases in which there is slight, or perhaps no appreciable affection of the joints, called rheumatic fever, are usually very refractory, and certainly still oftener prove fatal. No explanation of this curious fact, so far as I am aware, has heretofore been offered. Writers in noticing it, pass it over without any attempt of the kind. It has for some time occurred to me, that the violence and intractability of such attacks were owing to some internal lesion or lesions of a rheumatic nature, maintaining this indomitable fever, which is to be deemed merely an effect. Not having had any opportunities of autopsic inspections, I am not able to verify the conjecture by this test. But the tendency of the disease to a centripetal direction is well known. Bouillaud, as we have seen, pretends to have demonstrated, in a very large proportion of instances, the connection of the acute cardiac phlegmasiæ with articular rheumatism. Even admitting such a coexistence, which, for reasons previously stated, I cannot, to the extent claimed, it would be unsatisfactory in application to the present case. There is here scarcely ever any sensible affection of the joints; and those of the heart are so prominently displayed by severity of pain, and other symptoms, as not to be overlooked. The lesion must be in the mucous surface of the primæ viæ, or some other part, equally disposed to conceal its injuries by withholding the outward and visible signs of them.

Rheumatism, however, is one of those diseases, under all circumstances, exceedingly apt, on being confirmed, to run a tedious course of several weeks, and, when life is preserved, then gradually to slide into chronic degenerations, and especially the articular form of it.

From the preceding observations may be deduced the portents of the adverse events of the disease. The contrary, or indications of convalescence, are the subsidence of the local pain and inflammation, with the abatement, or total cessation of fever, a moist, soft, perspirable skin, open bowels, and copious discharges of urine, depositing a lateritious sediment.

Concerning the phenomena on dissection in acute rheumatism, these are not well understood; death, as just said, seldom happening to afford the means of inspection. Yet we are not destitute of intelligence on the subject. Examinations have shown that, in the articular form, the structure of the joint is uniformly affected in some degree. The synovial membrane, especially, undergoes considerable changes, being found injected, red, and swollen, with serous or sero-lymphatic, or sero-puriloid, or sanious effusions. Between the theca of the tendons, or rather within the surrounding cellular membrane, we meet with pretty nearly the same appearances. Extravasations of a peculiar gelatinous fluid are, however, more common events. The latter is the proper product of fibrous inflammation, which, at least, never ends in suppuration, or gangrene; and, when the other results take place, in articular rheumatism, they are to be referred to the textures mentioned.

In the muscular species, scarcely any appreciable alteration is usually discernible. But in very violent attacks, the affected muscle exhibits a reddish brown colour, and is so softened as readily to be torn, the interstitial membrane containing bloody serum, or pus, and sometimes even an abscess.

Those of the heart excepted, which have been accurately determined, the appearances in the other internal organs, I do not know, though it is presumable they are like the lesions of the external structures. The heart, itself, sometimes exhibits the characteristics of spasm, only, where death suddenly happens. But in more lingering cases, we are presented with all the phenomena of active phlogosis, in its substance, its fibro-serous envelope, and in the lining of its cavity, the one or the whole being implicated. The great arteries, the aorta, especially, have been discovered in similar conditions.

In many of its features it is so analogous to that of gout, the pathology of rheumatism need not detain us long. Consisting in inflammation of the fibrous or muscular tissue, to this most of its peculiarities may be assigned. That such is true, seems to be proved by the fact, that the disease originating in, or being translated to other parts, these are lost. Thus we see, when seizing on the mucous surface of the bowels or lungs, how striking is the difference! The eye affords a further conspicuous illustration to the same purport Composed of diverse tissues, each phlogosed has its own characteristics; the sclerotica alone, which is fibrous, presenting those of a rheumatic affection.

Located precisely as gout, there is, however, as I have indicated, considerable dissimilarity in the nature of the two diseases, which, I repeat, is partly to be ascribed to a want of identity in the causes whence they proceed, the variations of morbid action being as much influenced by this circumstance, as by the position it may occupy. Nor must we fail to advert to an additional consideration, which, perhaps, still more determines the discrepancies in these allied affections. Gout has its own distinctive diathesis, and whatever operates on it necessarily gives rise to phenomena sui generis, on changing the affinity to any other condition.

But other views have been and are entertained of the pathology of rheumatism. The idle notions of antiquity regarding it may be pretermitted, and, indeed, the only one I deem worthy of attention, is that recently promulgated, which supposes rheumatism to be a neuralgic affection, proceeding mainly from spinal irritation. Embarrassed by the peculiarity of the rheumatic state, having then no precise knowledge of the influence of causes or textures in this respect, pathologists, as long ago as the period when it was so much the habit to seek shelter for ignorance under the term nervous, had refuge in this resource. But of late, the hypothesis has been presented in a more definitive and attractive shape. Having in the consideration of neuralgia pointed out the difference between it and rheumatism, I shall not now pass over the same ground of discussion. That neuritis, or neuralgia of the great nerves, and especially the sciatic, has been too frequently mistaken for rheumatism of the adjacent muscles, or certain spinal irritations, confounded with lumbago, or affection of those of the small of the back, I am not now to be told. But further than this, I cannot admit, and must here recur to a remark formerly made, that no reason can be shown why the fibrous and muscular tissue may not be primarily affected, as well as any other of those of the body.

(*To be continued.*)

